# Characteristics of ketoacidosis diabetic at Fatmawati Hospital

**DOI:** 10.1186/1687-9856-2013-S1-P2

**Published:** 2013-10-03

**Authors:** Bina Akura, Ibnu Sastrawigoena

**Affiliations:** 1 Pediatric endocrinology division, Fatmawati hospital, Jakarta, Indonesia; 2 Registrar of pediatric department, Fatmawati hospital, Jakarta, Indonesia

## Background

Diabetic ketoacidosis (DKA) is the most common complication of diabetes mellitus. DKA results from absolute or relative deficiency of circulating insulin and from combined effects of increased counter regulatory hormone levels. The combination of low serum insulin and high counter regulatory hormone concentrations accelerate catabolic state with increased glucose production by liver and kidneys (by glycogenolysis and gluconeogenesis), impair peripheral glucose utilization causing hyperglycemia and hyperosmolality, and increase lipolysis and ketogenesis, resulting in ketonemia and metabolic acidosis. Most of Indonesian children with diabetes mellitus (DM) came for the first time with diabetic ketoacidosis.

## Aims

To describe the characteristics of diabetes mellitus type 1 cases presenting with ketoacidosis symptoms at fatmawati hospital.

## Methods

A retrospective survey was conducted during June 2012. Data was collected by using questionnaire. Subjects with ketoacidosis complication were included.

## Results

Of 20 children with diabetes mellitus type 1 at fatmawati hospital, only 13 subjects were included. Subjects were included had ketoacidosis event. Ratio between male to female almost equal (male 46.2% vs female 53.8%). Most subjects were 5-9 years old (46.1%) and 10-14 years old (38.4%). All subjects were not obese (100%) with body weight are 28.9±13.8 kg and body height are 134.8±16.4 cm. Most subject were severe ketoacidosis (53.8%) and small subjects were mild (23.1%), moderate (23.1%). Median glucose concentration is 339 (203-577)mg/dL and keton concentraton is 2.88±1.0 mg/dL. Hemoglobin A1c (HbA1c) is 11.1±1.9 % from all subjects. All subjects survived.

## Conclusions

Subjects were almost equal between male to female. Most subjects were aged 5-9 years old. Most subjects were severe ketoacidosis with median glucose concentration 339 (203-577) mg/dL and keton concentration 2.88±1.0 mg/dL. Most subjects were not obese with HbA1c 11.1±1.9%.

